# Pulmonary diseases induced by ambient ultrafine and engineered nanoparticles in twenty-first century

**DOI:** 10.1093/nsr/nww064

**Published:** 2016-10-08

**Authors:** Tian Xia, Yifang Zhu, Lina Mu, Zuo-Feng Zhang, Sijin Liu

**Affiliations:** 1 State Key Laboratory of Environmental Chemistry and Ecotoxicology, Research Center for Eco-Environmental Sciences, Chinese Academy of Sciences, Beijing 100085, China; 2 Division of NanoMedicine, Department of Medicine, University of California Los Angeles, Los Angeles, CA 90034, USA; 3 Department of Environmental Health Sciences, Fielding School of Public Health, University of California Los Angeles, Los Angeles, CA 90095, USA; 4 Department of Epidemiology and Environmental Health, School of Public Health and Health Professions, University at Buffalo, SUNY, Buffalo, NY 14214, USA; 5 Department of Epidemiology, Fielding School of Public Health, University of California Los Angeles, Los Angeles, CA 90095, USA

**Keywords:** air pollution, particulate matter, pulmonary disease, ultrafine particle, nanoparticle

## Abstract

Air pollution is a severe threat to public health globally, affecting everyone in developed and developing countries alike. Among different air pollutants, particulate matter (PM), particularly combustion-produced fine PM (PM_2.5_) has been shown to play a major role in inducing various adverse health effects. Strong associations have been demonstrated by epidemiological and toxicological studies between increases in PM_2.5_ concentrations and premature mortality, cardiopulmonary diseases, asthma and allergic sensitization, and lung cancer. The mechanisms of PM-induced toxicological effects are related to their size, chemical composition, lung clearance and retention, cellular oxidative stress responses and pro-inflammatory effects locally and systemically. Particles in the ultrafine range (<100 nm), although they have the highest number counts, surface area and organic chemical content, are often overlooked due to insufficient monitoring and risk assessment. Yet, ample studies have demonstrated that ambient ultrafine particles have higher toxic potential compared with PM_2.5_. In addition, the rapid development of nanotechnology, bringing ever-increasing production of nanomaterials, has raised concerns about the potential human exposure and health impacts. All these add to the complexity of PM-induced health effects that largely remains to be determined, and mechanistic understanding on the toxicological effects of ambient ultrafine particles and nanomaterials will be the focus of studies in the near future.

## INTRODUCTION

Air pollution is a major global problem associated with human health in both developing and developed countries [1–8]. The most common sources of air pollution include particulate matter (PM), ozone, nitrogen dioxide and sulfur dioxide [1,3,5,6]. PM can be defined by their different size ranges, including coarse (<10 μm), fine (<2.5 μm) and ultrafine particles (UFPs, <100 nm) [4–6,9]. Because numerous studies have shown that fine PM_2.5_ particle levels can be linked to premature mortality and adverse health effects, PM_2.5_ levels are often used as a major surrogate for air pollution [1,3–6,9,10]. According to the World Health Organization (WHO) Ambient Air Pollution database that comprises urban air-quality data for about 1600 cities from 91 countries for the years 2008–13, almost 90% of the urban population endured a PM_2.5_ concentration exceeding the annual mean values of 10 μg/m^3^ in the WHO air-quality guidelines [6]. In addition, WHO reported that, in 2012, around 7 million people died—one in eight of the total global deaths—as a result of air pollution exposure [5]. The new data revealed a stronger link between both indoor and outdoor air pollution exposure and the development of respiratory diseases, including acute respiratory infections, chronic obstructive pulmonary disease (COPD) and lung cancer, as well as cardiovascular diseases such as strokes and ischemic heart disease [5,6,11]. This finding further confirmed that air pollution is now the world's largest single environmental health risk factor and reducing air pollution could save millions of lives.

Globally, we are witnessing two opposing trends in terms of changes in the air pollution levels in different countries. Using new high-resolution global satellite maps of air quality indicators, NASA scientists tracked air pollution trends from 2005 to 2014 in various regions and 195 cities globally [12]. According to the research findings, the USA, Europe and Japan have improved air quality owing to strict emission-control regulations. As a result, in the USA, reductions in PM_2.5_ were associated with lower premature mortality and improvement in life expectancy. Long-term improvements in air quality were associated with statistically and clinically significant positive effects on lung-function growth in children [13]. On the contrary, China, India and the Middle East countries, with their rapid population growth, fast-growing economies and vastly expanding industrialization, have shown alarming increases in air pollution, along with increased mortality and disease burden in those countries [14]. On a positive note, in recent years, China's overall air quality has seen early signs of improvement due to a decrease in coal consumption, increase in renewable energy sources and stricter emission control. It is worth noting that, although air pollution mostly is considered a local or regional problem, studies have shown that air pollution could be transported over long distances, even across continents, in the troposphere [15]. Thus, mitigation of local air pollution could have far-reaching health benefits and a collective effort between every country involved would be important.

Despite the progress in our understanding of air pollution-induced diseases, much research remains to be done [1,8,9,14,16]. For instance, among the PM fractions, UFPs possess the highest particle number and surface area, carrying higher chemical contents than PM_2.5_ [4,17–20]. Studies have shown that UFPs have detrimental effects on both the respiratory and cardiovascular systems, and exacerbation of asthma [2–4,17,21,22]. However, our understanding of UFPs is still incomplete because of a deficiency in extensive UFP-monitoring networks, rapid physicochemical characterization techniques, and limited epidemiological and toxicological studies. In addition, the rapid advances in nanotechnology lead to production of high volumes of nanomaterials and increasing use in commercial products [18,23,24]. The use of nanomaterials increases the potential for human exposure to nanomaterials that could generate adverse health effects. For instance, ceria (cerium dioxide) are used in diesel as a catalyst to promote the combustion process, which are released in the diesel exhaust, potentially leading to lung exposure. The addition of ceria to diesel fuel resulted in ceria-concentration-dependent emission reductions of CO_2_, CO, total particulate mass, formaldehyde, acetaldehyde, acrolein and several polycyclic aromatic hydrocarbons; however, it also led to decreases in the size of emitted particles and a substantial increase in the number of UFPs (+32%), together with increases in certain other air pollutants, specifically NOx (+9.3%) and the particle-phase benzo[a]pyrene toxic equivalence quotient (+35%) [25]. This shows the complexity of the potential impacts introduced by nanomaterials. Given the health concerns related to UFPs and increasingly produced and used nanoparticles, further research is needed to evaluate the health risks associated with these tiny particles.

## EPIDEMIOLOGICAL STUDY LINKING PM TO PREMATURE MORTALITY

**Figure 1.**
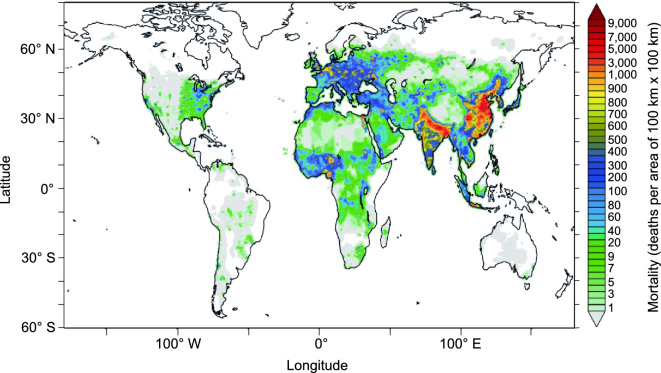
The contribution of outdoor air pollution sources to premature mortality on a global scale. The data were derived from a global atmospheric chemistry model that links premature mortality to outdoor air pollution, mostly by PM_2.5_. Study found that, in 2010, outdoor air pollution, mostly by PM_2.5_, leads to 3.3 million premature deaths per year worldwide, predominantly in Asia. Unit of mortality was expressed as deaths per area of 100 km × 100 km (color-coded). In the white areas, annual mean PM_2.5_ and O_3_ are below the concentration–response thresholds where no excess mortality is expected. The figure was originally published by [14] and has been approved for reuse by Nature Publishing Group.

Evidence that links ambient PM to a decrease in life expectancy and increase in premature mortality came from epidemiological studies that have been extensively reviewed before [1,7,8,24,26–28]. In a recent study, Chen *et al.* presented findings that implicated long-term exposure to air pollution particles contributed to enormous loss of life expectancy in China [26]. These results were based on an experimental design making use of a Chinese policy that provided free coal for heating in cities located north of Huai River, but not in the south, which produced an arbitrary discontinuity for PM air pollution, where the major difference was coal combustion. As a result, mean life expectancy is about 5.5 years (95% conficence interval (CI): 0.8, 10.2) lower in northern compared with southern China due to an increased incidence of cardiorespiratory mortality [26]. This finding correlated well with a study by Pope *et al.* that used a temporal difference in PM levels observed since the 1990s, when air quality across cities in the USA improved substantially. They found that there was an association between reductions in PM_2.5_ and an increase in life expectancy; a reduction of 10 μg/m^3^ was associated with an increase of 0.61 years in life expectancy [8]. There was also a strong evidence base for morbidity and mortality associated with both short-term (days to weeks) and long-term (years to decades) PM exposures. Early evidence linking ambient PM to mortality came from well-documented short-term extreme air pollution episodes (that lasted for days) in the 1930s to 1950s [28]. More recently, numerous daily time-series and case-crossover studies have observed a small but statistically robust relationship between daily mortality and short-term (days to weeks) elevation in PM [28]. In addition, short-term air pollution exposure could also increase the mortality rate of patients with respiratory diseases. For example, Cui *et al.* evaluated air pollution using the air pollution index (API) derived from the concentrations of particulate matter, sulfur dioxide, nitrogen dioxide, carbon monoxide and ground-level ozone and their relationship with the case fatality of severe acute respiratory syndrome (SARS) in China [29]. Case fatalities of patients from regions with high APIs (API > 100) and moderate APIs (75–100) were compared with that of patients from regions with low APIs (API < 75). The study showed that the case-fatality rate increased with the increment of API (case fatality = –0.063 + 0.001 × API). The correlation coefficient between API and SARS fatality was 0.8568 (*P* = 0.0636) [29]. Short-term exposure demonstrated that SARS patients from regions with moderate APIs had an 84% increased risk of dying from SARS compared with those from regions with low APIs (relative risk (RR) = 1.84, 95% CI: 1.41–2.40). Similarly, SARS patients from regions with high APIs were twice as likely to die from SARS compared with those from regions with low APIs (RR = 2.18, 95% CI: 1.31–3.65). For long-term studies, two large-scale prospective cohort studies in the USA showed that there were statistically robust associations between mortality risk and PM_2.5_ exposure even after smoking and other risk factors were controlled for [8,27]. Long-term air pollution exposure could also increase the mortality rate of patients with respiratory diseases such as SARS [29]. Although ecologic fallacy and uncontrolled confounding effects might have biased the results, the possibility of an effect of air pollution on the prognosis of SARS patients was indicated [29]. In a recent study, Lelieveld *et al.* used a global atmospheric chemistry model to investigate the link between premature mortality and ambient PM_2.5_ concentrations [14]. The authors found that more than 3.2 million deaths per year could be attributed to outdoor PM_2.5_ exposure. The majority of the mortality happened in Asia, which strongly influenced the global mortality rate. The highest number of deaths was in the Western Pacific, where China was the main contributor (1.36 million per year). Southeast Asia had the second highest premature mortality, where India was the main contributor (0.65 million per year) (Fig. 1) [14]. This was in addition to the estimated 3.54 million deaths per year caused by indoor air pollution resulting from biomass or coal combustion for cooking and heating [14]. The cause of premature mortality includes lung diseases, such as Chronic obstructive pulmonary disease (COPD), lung infection and cancer (described in detail below), as well as cardiovascular diseases and cerebrovascular diseases, etc. [14]. In addition, many birth cohort studies have linked PM_2.5_ exposure to asthma and allergic diseases [3,30]. Altogether, a large amount of literature provided evidence that breathing combustion-related PM_2.5_, even at exposure levels common to urban populations worldwide, contributed to cardiorespiratory disease mortality and diminished the lifespan [1,7,8,14,28]. There was also encouraging evidence showing that improvement in air quality benefited human health and increased the lifespan [8,27]. For example, long-term improvements in air quality were associated with a significant improvement in lung function in children [13]. However, there are no conclusive data yet available for the human health impacts of ambient ultrafine particles and engineered nanoparticles, which warrant further studies.

## AIR POLLUTION PARTICLE-INDUCED PULMONARY DISEASES

**Figure 2.**
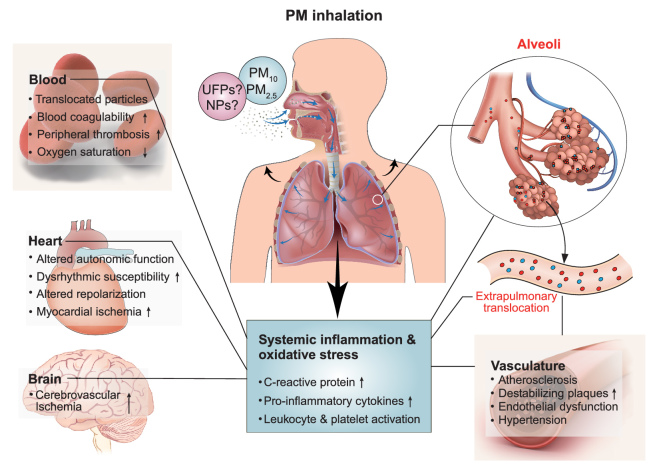
General toxicological pathways linking PM lung exposure to cardiovascular and cerebrovascular diseases that cause morbidity and mortality. The first line of defense against PM is the lung, where PM can induce or exacerbate lung diseases including COPD, asthma, lung infection disease and lung cancer. Furthermore, UFPs could translocate out of the lung into the blood stream and can cause systemic inflammation and oxidative stress that negatively impact blood and blood vessel, heart function and brain.

The lung is the first target organ for air pollution and PM exposure is associated with reduced lung function, increased lung inflammation, asthma, respiratory infections, lung cancer and exacerbation of COPD, which lead to systemic inflammation and oxidative stress affecting blood, vasculature, heart and brain, ultimately contribute to the premature mortality (Fig. 2) [3,8,14,16]. It is notable that the acute adverse health effects of PM are most often found in susceptible populations, including children, elderly people and those with chronic diseases [3,7], while not obvious for short-term exposure in normal healthy people except at very high concentrations [17]. However, air pollution exposure could induce adverse health effects to normal healthy people [17]. A panel study conducted among Chinese healthy adults during the Beijing Olympics found that peak expiratory flow levels increased in 78% of the participants when compared with the during- and pre-Olympics time points, while peak expiratory flow levels decreased in 80% of participants for the post- and during-Olympic periods comparison [31]. Children are more susceptible to air pollution because they have smaller airways, higher breathing rates per body mass, immature detoxification and metabolic systems, and they are more frequently exposed to outdoor air [3,10]. The elderly population and people with chronic diseases are susceptible likely because of less efficient particle clearance in the lung or impairment of immune functions. The adverse effects induced by air pollution are not limited to the lung, PM could induce systemic inflammation and oxidative stress, and nano-sized particles could even translocate out of the lung to other tissues and organs, which induces pathological changes in blood, vasculature, heart and brain (Fig. 4) [17,23]. The main pulmonary diseases that are associated with PMs are the following.

### COPD

COPD is not only associated with smoking, but also has high incidence among non-smokers [3,32,33]. The risk factors for COPD among non-smokers include indoor air pollution from biomass combustion and second-hand tobacco smoke, as well as occupational exposures and outdoor air pollution [32–34]. Epidemiological studies from both developing and developed countries have shown the association between outdoor PM exposure and COPD. In addition to PM, ozone and NO_2_ could also exacerbate COPD. An increase in ambient PM_2.5_ could induce acute exacerbations and mortality from COPD, while improvement in air quality decreases the incidence of COPD [32–34].

### Asthma

Abundant evidence has shown that air pollution could induce and exacerbate asthma [35]. In a recent meta-analysis of several birth cohort studies, the authors suggested that early childhood exposure (1–12 years old) to traffic-related air pollution (TRAP) containing UFPs and PM_2.5_ were associated with increased incidence of asthma and risk of sensitization to common allergens [30]. Furthermore, a 10-year longitudinal study of 23,704 adults from eight European countries showed an association between TRAP exposure and increased incidence of asthma [36]. The authors found that adult asthma incidence was positively associated with exposure metrics, including PM_10_, PM_2.5_, nitrogen oxides, traffic load and intensity. Further research with improved personal-level exposure assessment (vs. residential exposure assessment only) and phenotypic characterization was recommended by the authors [36].

### Lung cancer

In October 2013, the International Agency for Research on Cancer (IARC) of WHO announced that outdoor air pollution and PM were classified as a Group I carcinogen, which was determined based on the most recent data from human, animal and mechanistic studies [11]. In one of the first studies of lung cancer among female non-smokers in China with measured indoor exposure to PM_1_, PM_2.5_, PM_7_, PM_10_ and total suspended particulate (TSP), Mu *et al.* reported that the PM_1_ levels in cases were more than three times higher than those in the controls. Every 10 μg/m^3^ increase in PM_1_ is associated with a 45% increased risk of lung cancer [37]. Multiple studies in recent years have shown a correlation between air pollutants including PM_2.5_ and NO_2_ and lung cancer risk [3,38–40]. In 2010 alone, 223,000 deaths from lung cancer worldwide were associated with air pollution [11].

### Lung infections

Recent data showed that air pollution was associated with respiratory infections, due to the impaired immune functions of the lung and the susceptible population including children, elderly people and those with chronic diseases [41,42]. Two recent epidemiological studies demonstrated associations between air pollution (TRAP and ozone) and acute respiratory infections as well as increased emergency department visits in young children [41,42].

## PHYSICOCHEMICAL PROPERTIES OF AIR POLLUTION PARTICLES

**Figure 3.**
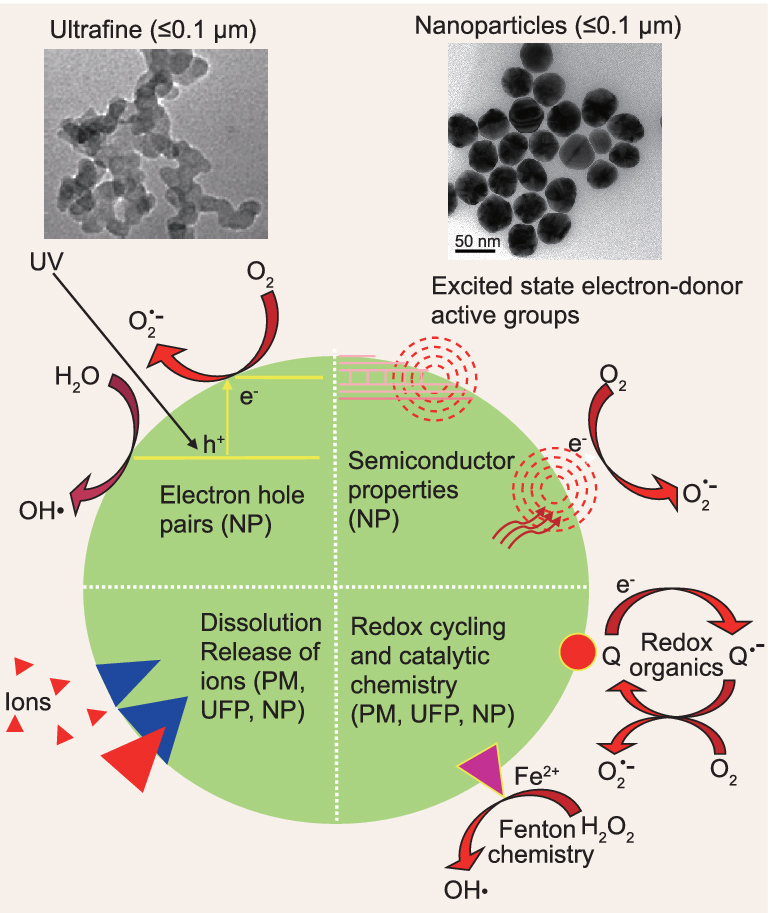
TEM images of ambient ultrafine particles and engineered nanoparticle as well as particle characteristics that promote or contribute to ROS generation. (a) Photoactivation effects of NPs, such as the formation of electron–hole pairs during ultraviolet exposure of TiO_2_; this effect has been associated with the generation of oxidative stress and inflammation by TiO_2_; (b) discontinuous crystal planes and material defects of NPs that lead to oxygen radical generation due to the active electronic state of the material surface; (c) redox cycling contributes to ROS production. This can occur due to the presence of transition metals or redox-cycling organic chemicals on the PM, UFP and NP surfaces. PMs and UFPs, for example, contain organic compounds such as quinones, which can generate ROS through redox cycling. Moreover, transition metals can generate hydroxyl radicals through the Fenton reaction. The Fenton reaction is one of the mechanisms by which metal impurities on the CNT surface can induce ROS production. Finally, (d) particle dissolution (e.g. ZnO, CdSe, Cu) can produce free ions that are capable of inducing ROS generation and oxidative stress in cells. Metal fume fever is a real-world example of this toxicity, commonly for welders.

The most common sources of air pollution include PM, ozone, nitrogen dioxide and sulfur dioxide [3,7,14]. Among these components, PM has been shown to play a major role in human morbidity and mortality [3,7,14]; thus, we will focus our discussion on PM. PM comes from natural and anthropogenic activities and processes [3,4,7,14,24]. Anthropogenic sources of outdoor PM are high-temperature processes (e.g., welding, smelting), combustion (e.g., power generation, land traffic, residential and commercial heating and cooking), industrial and other processes, including agriculture, dust and biomass burning. Indoor PM sources are mostly biomass and coal combustion for cooking and heating purposes [3,4,7,14,24]. Although the physiochemical properties (chemical composition, metal content, etc.) of PM are different worldwide, they are all associated with adverse human health effects although at different potency levels [14]. PM_10_ is referred to the mass of particles collected with 50% efficiency for particles with an aerodynamic diameter equal to or less than 10 μm; it should be noted that all particles down to the ultrafine size range were collected [17]. At this size range, according to the International Standards Organization thoracic convention, the mass fraction of inhaled particles could penetrate beyond the larynx to the airways [23]. PM_2.5_ refers to the respirable fraction that also contains the ultrafine component, which penetrates to the unciliated regions of the lung and is now being considered worldwide as the standard [23]. Ultrafine particles are found to a large extent in urban air as both singlet and aggregated particles (Fig. 3), and indeed are the predominant particle type by number in urban PM_10_ and PM_2.5_, although they contribute insignificantly to mass [4,18,23,24]. PM from combustion processes characteristically has an elemental or organic carbon core carrying trace metals, sulfate, ammonium, and volatile and semi-volatile components [43,44]. The composition of combustion-generated PM usually depends on fuel type, burn conditions and atmospheric conditions. Compared with PM_10_ and PM_2.5_, traffic-derived UFPs are challenging to characterize geographically or spatiotemporally, as their concentrations decrease sharply downwind from sources, and UFPs shift in size from nucleation mode to accumulation mode with time and distance from their emission point due to agglomeration and condensation [45–47]. For combustion sources, the fuel, combustion conditions and pollution controls will alter the particle numbers and size distribution of the PM emitted [43,44]. However, studies have shown that UFPs carried more organic chemicals due to their significantly larger surface area; many of these compounds were redox-active and had the ability to generate reactive oxygen species (ROS) [43,44]. Studies have shown that UFPs were more toxic than their larger counterparts; however, more research is needed to clarify the role of UFPs [17,21,22]. Nanoparticles (NPs) are intentionally created with specific size, shape, surface characteristics and functionality that are required for their applications [18,23]. There are similarities between NPs and UFPs; however, there are also major differences (Table 1). NPs and PM including UFPs could both generate ROS or release toxic ions through dissolution, through similar or different mechanisms (Fig. 3) [18,24,48]. Although currently there is no definitive evidence to link NP exposure to any human disease, much experimental data indicate that some NPs with certain physicochemical properties may be potentially hazardous [19,49–59]. Obviously, more research is needed on this front.

**Table 1.** Comparison of ambient PM_2.5_, UFPs and NPs.

Particle type	PM_2.5_	UFPs	NPs
Size	<2.5 μm, containing UFP component	<100 nm, exist alone or as a component of PM_2.5_	<100 nm for at least one dimension
Sources	Incidental (combustion)	Incidental (combustion)	Engineered (controlled synthesis)
Morphology	Irregular (chain-like structure)	Irregular (chain-like structure)	Regular (sphere, tube, cube, rod, wire, plate, etc.)
Homogeneity	No	No	Yes
Organic chemical content	Low (compared with UFPs)	High (compared with PM_2.5_)	None to very low levels
Metal impurity	High	High	Varies
ROS generation	Yes	Yes	Varies
Exposure route	Inhalation	Inhalation	Inhalation, dermal, ingestion, injection
Adverse health effects	Yes	Yes	Largely unknown

## DIFFERENT DEPOSITION PROFILES AND HAZARD POTENTIALS OF PARTICLES WITH DIFFERENT SIZES

**Figure 4.**
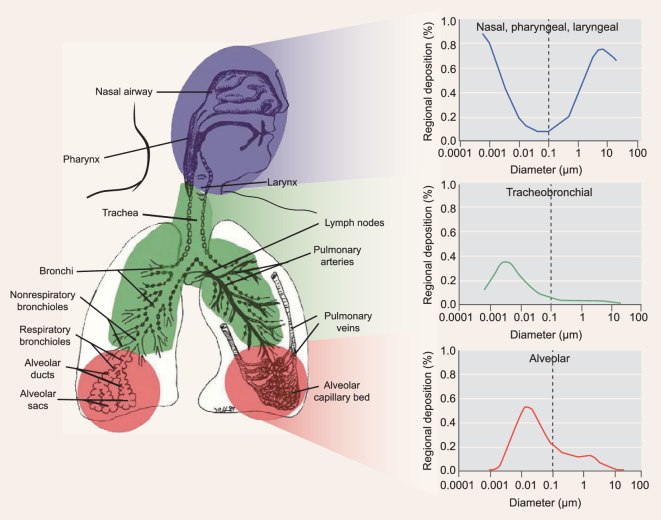
Predicted fractional deposition of inhaled particles in the nasopharyngeal and laryngeal, tracheobronchial and alveolar regions of the human respiratory tract during nose breathing. Please note the clear changes in the deposition rate at these lung regions between nano-sized particles and their larger counterparts. The figure was originally published by [23].

The potency of PM in causing an adverse health impact is dependent, in part, on its deposition in the airways and chemical composition [17,23]. The main deposition mechanisms in the respiratory tract for inhaled PM include impaction, sedimentation, interception and diffusion [23]. PM in different size ranges has drastic differences in distribution and deposition in the lung [23]. This is clearly demonstrated in Fig. 4, which shows the differential deposition of inhaled particles with different sizes in the nasopharyngeal, tracheobronchial and alveolar regions of the human respiratory tract [23]. For UFPs and NPs that are in the size range of 1–100 nm, the particles could penetrate deeper into the alveolar region and deposit there at high percentages. For larger particles including PM_10_ and PM_2.5_, they generally deposit in the nasopharyngeal and laryngeal region and poorly deposit in the alveolar region (Fig. 4) [23].

**Figure 5.**
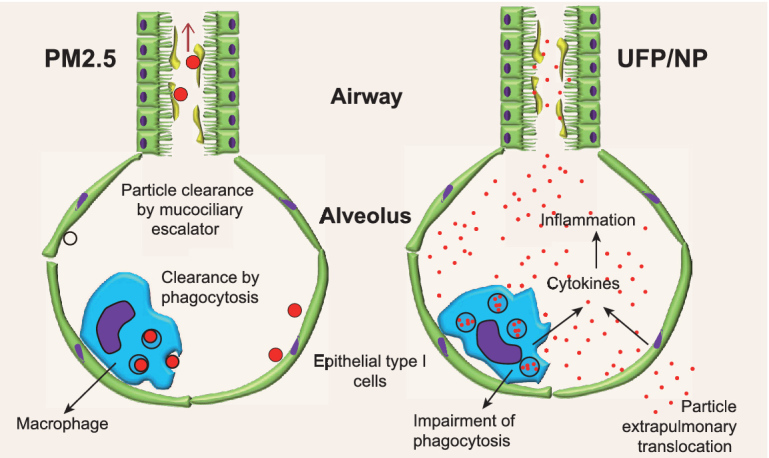
Scheme for physicological and toxicological events occurring after PM_2.5_ and UFP/NP exposure. PM_2.5_ mostly sticks to the airway, where they are cleared by mucociliary escalator. The remaining PM_2.5_ particles that get inside the alveoli will be phagocytosed by resident macrophages. However, for UFPs and NPs, the majority of the particles will get inside the alveoli, where the cellular uptake of nano-sized particles by macrophages is impaired, leading to delayed clearance of particles and generating oxidative stress and pro-inflammatory effects by macrophages and epithelial cells because of prolonged exposure to particles. The prolonged interaction between particles and the epithelium also lead to extrapulmonary translocation to the lung interstitium and even into the circulation. Adapted from [17].

The different deposition profiles among particles with diverse sizes could at least partially explain their different health effects [17,23]. For example, the deposition site determines the clearance rate of particles [17,23]. For large particles including PM_10_ and PM_2.5_, the majority deposited in the upper airways could be removed with the mucus via the mucociliary escalator (Fig. 5). The small fraction of particles that could pass through upper airways into the alveolar region (Fig. 4), such as PM_2.5_ fine particles, could be easily endocytosed and removed by alveolar macrophages (Fig. 5). However, for UFPs and NPs, the majority of these particles could pass through the airways into the alveolar region. Although they also deposit in the larger airways, this only accounts for a small percentage of the total number of particles in the lung. Furthermore, the particles are so tiny and in such vast numbers that macrophages could not take them up effectively (Fig. 5) [17,23]. The interactions among particles, epithelial cells and macrophages could generate oxidative stress and pro-inflammatory effects in the lung, and there are reports showing that nano-sized UFPs could be more toxic than their larger counterparts (which will be discussed in later sections) [2,17,21,23]. In addition, extrapulmonary translocation across the epithelium could occur because of the reduced removal rate and longer retention of UFPs and NPs that allows transcytosis of these nano-sized particles [17,23]. This could lead to secondary deposition of these particles in other tissues and organs, which may contribute to further adverse health effects (Fig. 5) [17,23].

## OXIDATIVE STRESS PLAYS A MAJOR ROLE IN PM-INDUCED PULMONARY DISEASES

**Figure 6.**
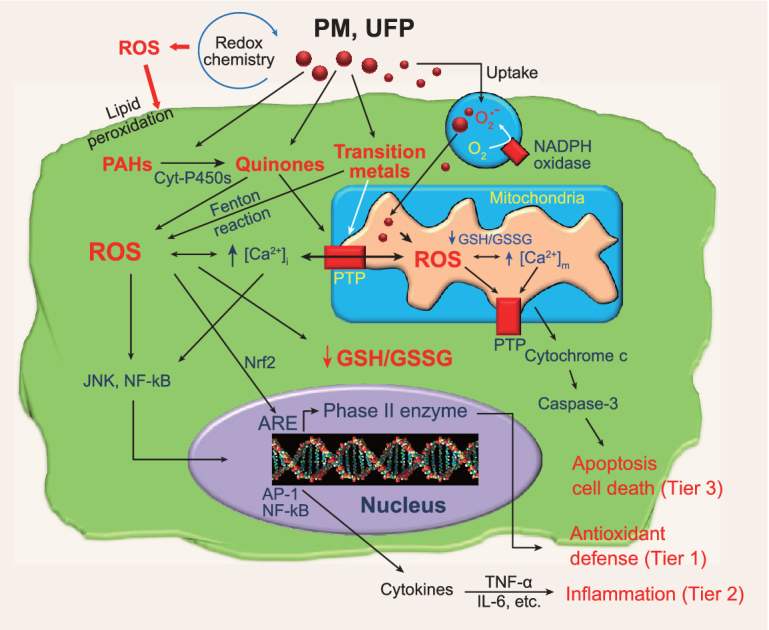
Sources of PM- and UFP-induced ROS production and their cellular effects. Quinones, under the catalytic influence of NADPH-cytochrome P450 reductase, can redox cycle to produce ROS in the endoplasmic reticulum. Phagocytosis can induce the assembly and activation of NADPH oxidase to produce superoxide. UFPs can interfere in electron transduction in the mitochondrial inner membrane as well as perturb the PT pore to generate ROS. ROS induce lipid peroxidation in the cell membrane, cross-linking of protein thiol (SH) groups and DNA damage. ROS can also deplete glutathione (GSH), resulting in oxidative stress in the cell. Depending on the levels of oxidative stress, the response could range from induction of Nrf2 release to the nucleus, activation of MAPK and NF-kB signaling cascades, or cytotoxicity. According to the hierarchical oxidative stress hypothesis, Nrf2 interaction with the antioxidant response element (ARE) leads to heme oxygenase 1 and other phase II enzyme expression at lower levels of oxidative stress (Tier 1), while, at a intermediary level of oxidative stress, activation of the MAPK and NF-kB signaling cascades can induce pro-inflammatory responses (e.g. cytokine and chemokine production) (Tier 2). At the highest oxidative stress level (Tier 3), ROS can induce the opening of the mitochondrial PT pore, followed by cytochrome c release, caspase-3 activation and induction of programmed cell death.

**Figure 7.**
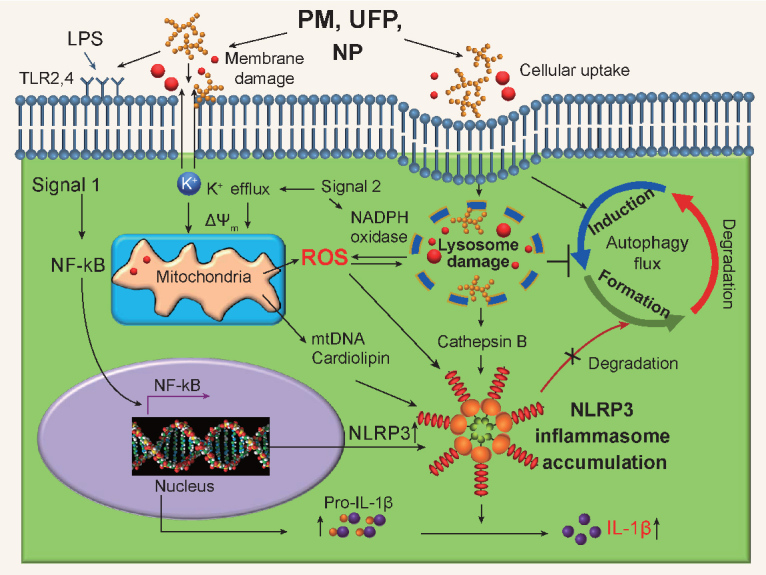
Potential mechanisms of PM-, UFP- and NP-induced NLRP3 inflammasome activation and IL-1β production. Activation of NLRP3 inflammasome complex can be induced by various stimuli including PM and NP particles, and the pro-IL-1β is processed by the inflammasomes to produce mature IL-1β, while IL-1β plays a major role in inducing the pro-inflammatory and pro-fibrogenic effects in the lung. NLRP3 inflammasome activation requires two signals *in vitro*. For signal 1, pathogen-associated molecular patterns (PAMPs) such as LPS is recognized by Toll-like receptor 4 (TLR4) residing on the cell membrane, which further leads to NF-κB activation and the production of pro-IL-1β and NLRP3 proteins. NLRP3 inflammasome activation mechanisms include ROS generation, potassium efflux, lysosomal damage and cathepsin B release. After phagocytosis of PM, UFPs and NPs, NADPH oxidase is activated to generate ROS. The over-production of ROS may cause the destabilization and permeabilization of lysosomes and cathepsin B release, which will initiate the inflammasome activation cascade. Mitochondrion is another important source of ROS in cells. Over-production of mitochondrial ROS could lead to release of mtDNA and cardiolipin that could induce NLRP3 inflammasome activation. In addition, potassium efflux induced by particles could also induce mitochondrial and cellular ROS production leading to NLRP3 inflammasome activation. Levels of activated NLRP3 inflammasomes are tightly regulated by autophagy.

Emerging evidence has shown that, among different particles, UFPs are potentially the most dangerous owing to their small size, deep penetration, large surface area/volume ratio, high content of redox-cycling organic chemicals, alveolar deposition and high rates of retention in the lung [4,21,22]. Compared with PM_10_ and PM_2.5_, traffic-derived UFPs are capable of carrying more organic chemicals on their significantly larger surface; many compounds (polycyclic aromatic hydrocarbons (PAHs) and quinones) are redox-active and have the ability to generate ROS [4,20–22,24,44,60]. While PM_10_ and PM_2.5_ can be easily removed by phagocytosis, the extremely small size of UFPs enables them to evade such a process and UFPs could have more interactions with other cell types in the lung (Fig. 5) [17,23]. These specific features of UFPs can significantly contribute to the adverse effects through ROS over-production by the redox-active organic chemicals and metals on particle surface, resulting in cellular oxidative stress [18,19,21,44,48]. Oxidative stress has been identified as a major mechanism for PM_10_-, PM_2.5_- and UFP-associated health effects, including exacerbation of asthma and COPD, and promotion of atherosclerosis [18,19,21,22,44,48]. Some NPs, including carbon nanotubes, silver nanoparticles, ZnO, CuO, etc., have also been shown to be able to generate ROS and oxidative stress [19,55,56,58,59,61]. At the cellular level, particle-induced oxidative stress can activate a cascade of signaling pathways that mediate the production of pro-inflammatory cytokines/chemokines and induce apoptosis (Figs 6 and 7) [18,48,53], resulting in inflammation and tissue injury in the respiratory and cardiovascular systems [17,33,38].

## MOLECULAR MECHANISMS OF PM-INDUCED OXIDATIVE STRESS AND TOXICITY

PM-induced ROS production in biological systems and targeted cells originates from a variety of sources (Fig. 6) [18–21,24,44,60]. These include: (i) carbon core of PM and UFPs could induce ROS generation and oxidative stress; (ii) catalytic conversion of PAHs to quinones by cytochrome P450 in the endoplasmic reticulum; (iii) quinone redox cycling by NADPH-dependent P450 reductase in microsomes; (iv) mitochondrial perturbation leading to electron leakage in the inner membrane; and (v) NADPH oxidase activity in the macrophage surface membrane and associated phagosomes. Both the carbon cores as well the chemicals coated on their surface play a role in these biological events [2,17,44]. This also includes the involvement of transition metals in generating ROS by a Fenton reaction [2,17,44].

The PM backbone is formed by chain-like carbon-based nanoparticles. To see whether the particles themselves could induce ROS and adverse effects, ultrafine carbon black was selected as the model particle. Studies found that ultrafine carbon black could generate ROS in cell-free systems and cause oxidative stress to cultured cells [2,17,23]. In addition, ultrafine carbon black caused systemic pro-inflammatory responses in the lung including modest neutrophil influx and protein leak [17,23]. There was evidence of systemic oxidative stress in the plasma and increased production of plasma factor VII, which is an independent risk factor for cardiovascular disease [17,23].

Redox-cycling organic chemicals on PM surfaces, such as quinones, are capable of generating ROS in cellular targets such as bronchial epithelial cells, macrophages and endothelial cells [24,44,60]. Quinones are byproducts of fossil fuel combustion, as well as the enzymatic conversion of PAH in the lung. Redox-cycling quinones undergo one-electron reductions mediated by NADPH-cytochrome P450 reductase to form semiquinones. These semiquinones are metastable and donate electrons to O_2_, leading to the formation of O_2_$^{\scriptstyle\bullet}$^−^. Due to their high content of organic chemicals, ambient UFPs contribute proportionally more redox-cycling chemicals than larger particles [4,24,44,60].

Catalytically active metals adsorbed on the PM surface have been shown to contribute to oxidative stress *in vitro* and *in vivo* [4,24,60]. PM contains a number of transition metals (coarse > fine > UFP) that contribute to ROS production. Among the transition metals, Fe, Al, Cu, Ni, Mn, Zn, Cr, Ba and Sr are the most abundant [44]. In the presence of hydrogen peroxide, some of these metals, including Fe^2+^, have the ability to generate the hydroxyl radical ($^{\scriptstyle\bullet}$OH) through catalysis of a Fenton reaction:

\[
{\rm{F}}{{\rm{e}}^{2 + }} + {{\rm{H}}_2}{{\rm{O}}_2} \to {\rm{F}}{{\rm{e}}^{3 + }}{ + ^ \bullet }{\rm{OH}} + {\rm{O}}{{\rm{H}}^{-}}.
\]

The $^{\scriptstyle\bullet}$OH radical is more reactive than O_2_$^{\scriptstyle\bullet}$^−^ and hydrogen peroxide by several orders of magnitude. Besides ROS generation, transition metals also have the ability to directly perturb the function of the mitochondrial permeability transition (PT) pore [24,44,60]. Transition metals may also act synergistically with other PM components in impacting mitochondrial function, ROS generation, ATP production and cell viability [24,44,60].

PM components could perturb mitochondrial function, leading to generation of ROS and cell death [21,60]. Mitochondria have been shown to be a direct subcellular target for ambient UFPs. For example, ambient UFPs have been found to lodge in the mitochondria of target cells and cause mitochondrial damage [21]. Although the mechanism of mitochondrial localization is still elusive, we hypothesize that particle size, hydrophobicity and presence of organic chemicals may play a role in the process. In addition to direct effects, studies have found that organic PM chemicals are capable of generating ROS by their ability to interfere in these electron transfer events. This includes the effect of polar chemicals such as redox-cycling quinones to disrupt electron transfer in the inner membrane [44]. This disruption in electron flow could favor the formation of ubisemiquinones, thereby contributing to mitochondrial O_2_$^{\scriptstyle\bullet}$^−^ production. In addition, organic chemicals such as quinones and PAHs also have the capability to perturb the mitochondrial PT pore (Fig. 6) [44,60]. The PT pore is a redox-, pH-, calcium- and ΔΨ_m_-dependent protein complex, which plays a pivotal role in regulating mitochondrial function and controlling cellular apoptosis. Opening of the PT pore could lead to mitochondrial swelling and rupture of the mitochondrial outer membrane, by which various pro-apoptotic proteins such as cytochrome c, SMAC, apoptosis-inducing factor (AIF), etc., are released into the cytosol where they activate a number of apoptotic pathways, ultimately leading to programmed cell death [18,24,44,48,60,62].

PM-, UFP- and NP-induced oxygen radical generation can result in cellular and tissue injury responses such as inflammation, apoptosis, necrosis, fibrosis and carcinogenesis [18,20,48,63]. Cellular oxidative stress can generate a wide range of responses that can be experimentally detected, including damaged DNA—mainly DNA single-strand breaks or generating 8-oxo-2΄-deoxyguanosine (8-oxo-dG) that promote cell turnover and proliferation [63]. At the lowest level of oxidative stress (Tier 1), the induction of antioxidant and protective responses is mediated by the transcription factor Nrf2, which regulates the activation of the antioxidant response element in the promoters of phase II genes [18,64]. As the levels of oxidative stress increases (Tier 2), this protective response may yield to pro-inflammatory responses because ROS induces redox-sensitive signaling pathways such as the mitogen-activated protein kinase (MAPK) and nuclear factor-kappa B (NF-κB) cascades [18,20,24,53,60]. At the highest level of oxidative stress (Tier 3), a perturbation of mitochondrial inner membrane electron transfer and the open/close status of the PT pore can trigger cellular apoptosis and cytotoxicity. This outcome is also known as toxic oxidative stress (Fig. 6) [18,20,24,53,60]. Using this three-tier screening platform, we have also shown for ambient air-pollution particles that one can link the hierarchical oxidative stress paradigm to the *in vivo* outcomes in animal disease models [18,20,24,53,60]. Oxidative stress is also involved in mutagenesis [65]. Future screening could also include DNA damage and mutagenesis tests to assess their carcinogenic potential.

## HAZARD POTENTIAL OF NANOPARTICLES IS DETERMINED BY THEIR PHYSICOCHEMICAL PROPERTIES

NPs are intentionally created with specific size, shape, surface characteristics and functionality that are required for their applications [18,23,24,48,53,66]. Although currently there is no definitive evidence to link NP exposure to any human disease, experimental data indicate that many NPs may be potentially hazardous [19,49–52,54–56,58,59,61,67,68]. The physicochemical characteristics of NPs that may have health implications include particle size, shape, aspect ratio, composition, surface reactivity, solubility and ability to generate ROS [18,48,53]. Similar to UFPs, the nano-scale size can enhance NP translocation and deposition by interfering with their clearance [17,23]. NP composition or modification may affect particle surface reactivity such as the ability to generate ROS, which is an important mediator involved in various human diseases. However, UFPs and NPs are inherently different in many aspects, such as source, morphology, organic chemical content, homogeneity, ROS production, possible exposure route and the potential adverse health effects (Table 1). Extensive *in vitro* and *in vivo* studies have been performed to elucidate the toxic effect of engineered nanomaterials (ENMs) and physicochemical properties of ENMs, including morphology, size, dissolution, aspect ratio, surface coating, surface reactivity, bandgap and aggregation, have been suggested to determine their toxicity potentials [17,18,23,48,53]. For example, CeO_2_ has excellent antioxidant properties that are enabled by the duality of the cerium ion to easily cycle between Ce^4+^ and Ce^3+^; it is widely used as a catalyst or fuel additive in diesel to facilitate the combustion process and is released into the air as exhaust. There are reports over the past few years that utilize the antioxidant property of CeO_2_ in treating diseases such as cancer, Alzheimer's, cardiac arrest, radiation-induced cell death and aging [69]. However, a recent study found that CeO_2_ NPs could lead to a decrease in cell viability and the treated cells exhibited characteristic hallmarks of apoptosis [70]. In this case, CeO_2_ NP toxicity is caused by mitochondrial damage leading to AIF release, but not caspase activation or ROS production. Moreover, CeO_2_ NP exposure leads to autophagy and inhibition of autophagy partially reverses cell death by CeO_2_ NPs [70]. Obviously, more research should be done to clarify the effects of CeO_2_ under *in vitro* and *in vivo* conditions.

TiO_2_ is the most widely produced nanomaterial and is found in cosmetics, sunscreen, paint, vitamins, toothpaste, food colorants, nutritional supplements, etc. However, study has shown that TiO_2_ nanoparticles could cause oxidative stress-mediated acute lung inflammation. Oberdorster *et al.* have shown that, on a mass-dose basis, ultrafine TiO_2_ is more toxic than fine TiO_2_ [23]. However, when the doses of particles were expressed in forms of particle surface area, the responses of ultrafine and fine TiO_2_ particles fell on the same dose–response curve. This suggests that surface area is an important property for NPs when considering their toxic potential. Also, there is a study showing that TiO_2_ in anatase form was more potent in ROS generation than in rutile form, revealing the important role of crystal structure [23]. Under low levels of ultraviolet light, TiO_2_ nanoparticles could generate ROS and induce cytotoxicity because of their photoactivation property [71]. Another example is ZnO nanoparticles, which have received significant attention due to their wide use in sunscreens, electronics, optics and photonics. However, pulmonary exposure of ZnO nanoparticles could lead to transient increases in acute lung inflammation, similarly to a disease called metal fume fever. It has been shown that the toxicity of ZnO is dependent on the particle dissolution property and shedding of toxic Zn ions, which induce oxidative stress and pro-inflammatory effects *in vitro* and *in vivo* [58,68,71].

In addition to oxidative stress paradigm, recently studies have found Nod-like receptor protein 3 (NLRP3) inflammasome activation plays a major role in PM- and NP-induced chronic effects such as lung fibrosis [49–51,55,56,61,72–75]. Nanomaterials including rare earth oxides (REOs), high-aspect-ratio materials including carbon nanotube (single-walled and multi-walled), TiO_2_ nanobelts and CeO_2_ nanorods, 2D materials including graphene and graphene oxide could induce NLRP3 inflammasome activation and a series of events leading to epithelial mesenchymal transition and lung fibrosis. Carbon nanotubes (CNTs) are a type of long-aspect-ratio nanomaterial which is drawing wide interest because of their potential applications in electronics, optics, drug delivery and cancer therapy. However, animal studies have shown that they could promote the production of pro-fibrogenic cytokines and growth factors (IL-1β, TGF-β and PDGF-AA, etc.) that may lead to lung fibrosis [72–75]. The mechanism involves cellular uptake of CNTs, over-produced ROS by NADPH oxidase activation, lysosomal damage induced by the surface reactivity of CNTs and the release of lysosomal protein, cathepsin B, which activates NLRP3 inflammasome and produces IL-1β. IL-1β plays a major role in inflammation and fibrosis by activating epithelial mesenchymal transition in the lung (Fig. 7) [56,72–75]. Wang *et al.* showed that the dispersal state, hydrophobicity and purity of multi-walled carbon nanotubes (MWCNTs) could affect the pro-fibrogenic cellular responses that also correlate with the extent of pulmonary fibrosis [72–75]. Furthermore, other long-aspect-ratio ENMs also showed similar effects. Ji *et al.* demonstrated the toxicological effects of long-aspect-ratio nanomaterials using a library of in-house-made CeO_2_ nanoparticles [76]. *In vitro* toxicity study demonstrated that, at lengths ≥200 nm and aspect ratios ≥22, CeO_2_ nanorods induced progressive pro-inflammatory effects and cytotoxicity [76]. The relatively low ‘critical’ length and aspect ratio were associated with small nanorod/nanowire diameters (6–10 nm), which facilitates the formation of stacking bundles that could pierce through cell membrane, causing the release of cathepsin B and further the activation of NLRP3 inflammasome. The same CeO_2_ nanorods could also induce significantly higher lung fibrosis than spherical particles *in vivo* [77]. Our recent studies show that rare earth oxide nanoparticles (REOs), which are widely used in electronics and upconversion nanoparticles for bio-imaging, are unstable in the acidic physiological environment, including the lysosomal compartment of the cells [49–51]. These REOs can dissolve and the dissolved ions could bind to phosphates strongly; as a result, the particles transform from spheres to ‘sea-urchin’-shaped or mesh-like structures with composition changes to the RePO_4_. The sources of phosphates are not limited to free phosphate ions in the lysosomal compartment; they also include phosphate groups on intracellular proteins and membranes. On the one hand, biotransformation of REOs could lead to dephosphorylation of phospholipids on the lysosomal membrane, which destabilizes the membrane, leading to lysosomal damage. Released cathepsin B from lysosomes to the cytosol will activate NLRP3 inflammasomes to produce IL-1β, which initiate a cascade of events that culminate in pulmonary fibrosis [24,26,27]. On the other hand, stripping of phosphate groups from lysosomal proteins will lead to loss of enzymatic activities of the proteins, resulting in lysosome dysfunction, which leads to lysosome and autophagosome fusion inhibition and compromised autophagosome degradation in the autophagy flux. This leads to accumulation of activated NLRP3 inflammasomes because autophagy is the major homeostatic mechanism to remove activated inflammasomes [51]. The above examples show that the physicochemical properties of NPs determine their toxic potential to human health, and more research is needed to understand these interactions between numerous other types of NPs and biological systems occurring at the nano–bio interface [18,48,53,66,78].

## PREDICTIVE TOXICOLOGICAL APPROACH TO STUDY AMBIENT ULTRAFINE PARTICLES AND NANOPARTICLES

Studies have shown that exposure to UFPs and NPs has the potential to cause adverse health effects in humans [18,21,22,24,48,53]. In order to assess the toxic effects of these nano-sized particles, we advocate a predictive toxicological approach with the goal of linking particle physicochemical properties to their toxic effects [18,48,53]. To establish this predictive toxicological paradigm, there are five major requirements. The first is to comprehensively characterize their physicochemical properties that may lead to biological injury. It also requires the establishment of nanomaterial libraries with property variations, which allows building the link between material property and the biological/toxicological activities. The second requirement is to develop *in vitro* cellular screening assays that reveal particle-induced injury mechanisms and pathways such as oxidative stress and NLRP3 inflammasome activation. Third, where possible, it is important to develop high-throughput screening platforms to assess the large number of material physicochemical properties, dosage and time points that are likely to lead to biological injury. Fourth, the *in vitro* data could be used for *in silico* modeling to establish hazard ranking and structural-activity relationships that can be used to predict ENM toxicity. Finally, the *in vitro* hazard ranking and structural-activity relationships will then need to be validated by limited *in vivo* animal experiments to establish the ‘predictiveness’ of this approach. We have successfully demonstrated the usefulness of the predictive toxicological approach for the hazard assessment of over 200 different nanomaterials and development of major structure activity relationships for groups of nanomaterials. We are confident that this approach would facilitate research on the toxicological effects induced by PMs, UFPs and NPs [53].

## CONCLUSIONS

Convincing evidence has established the association between PM and many pulmonary diseases that contribute to early mortality and reduced life expectancy. However, for ambient UFPs and NPs, although much progress has been made in understanding their toxicological effects and mechanisms of toxicity, there are still many knowledge gaps on their impact on human health. In this review, we have summarized recent major findings on cellular, animal and human research with a focus on the respiratory effects and mechanisms of toxicity induced by PM, UFPs and NPs. Available evidence strongly suggests that UFPs and NPs may be more potent in causing adverse health effects in humans because of their high deposition rate in the alveolar region, impaired clearance by alveolar macrophages and higher surface reactivity, pro-oxidative and pro-inflammatory effects than their larger counterparts. Thus, it is imperative to focus future research on the health effects of nano-scale pollutants so that preventive strategies and regulatory guidelines can be developed to reduce exposure and improve human health.
